# Morphology and Mechanical Properties of Polyamide 6/Polystyrene Blends Prepared by Diffusion and Subsequent Polymerization of Styrene in Polyamide 6 Pellets

**DOI:** 10.3390/ma11050776

**Published:** 2018-05-11

**Authors:** Yin-Le Tan, Cheng-Huan Huang, Zhao-Xia Guo, Jian Yu

**Affiliations:** Key Laboratory of Advanced Materials (MOE), Department of Chemical Engineering, Tsinghua University, Beijing 100084, China; tanyinle1992@hotmail.com (Y.-L.T.); hyhuangch@foxmail.com (C.-H.H.)

**Keywords:** nylon 6, polystyrene, polymer blends, mechanical properties, injection molding

## Abstract

Polyamide 6 (PA6)/polystyrene (PS) blend pellets were prepared by diffusion and subsequent polymerization of styrene in commercial PA6 pellets and processed into cuboid-shaped bars by injection molding. The average PS particle size in the bars was 240 nm, slightly higher than that in the blend pellets (120 nm), showing that only limited coalescence occurs during injection molding. The mechanical properties of PA6/PS bars were investigated by tensile, flexural, and notched impact tests. A 41% increase in notched impact strength was found without decreasing the modulus, tensile, and flexural strengths and elongation to break, when compared with those of neat PA6 bars. These good mechanical properties were attributed to the small PS particle sizes, and the good interfacial adhesion between PS particles and the PA6 matrix resulted from the occurrence of PS grafting onto PA6 during the preparation of the blend pellets and injection molding. The water sorption and water-induced dimensional changes in PA6/PS bars were significantly less than those of neat PA6 bars because of the presence of the hydrophobic PS phase. This work reveals that the PA6/PS quasi-nanoblend pellets are useful stock for plastic production.

## 1. Introduction

Polymer nanoblends are an important type of nanomaterial. They show superior properties compared to polymer microblends prepared by conventional melt blending [1,2,3,4,5,6,7,8,9,10,11]. Therefore, reducing the size of the dispersed phase of polymer blends is a very interesting topic in polymer science. Diffusion and subsequent polymerization of a monomer (or monomer mixture) in commercial polymer pellets is a method that has been explored in recent years by our research group for preparing polymer blend pellets with fine particle sizes down to the quasi-nanoscale [12,13,14,15,16,17,18,19]. It involves diffusion of a monomer (typically styrene) into the amorphous regions of semicrystalline polymer pellets and subsequent diffusion of an initiator (typically benzoyl peroxide) along the diffusion paths of the monomer. The particle size of the blend pellets is limited because of the confined polymerization. The blend pellets can be used directly in plastic production without the need for pre-compounding, and only a limited degree of particle coalescence occurs during processing, consequently improving the mechanical properties. So far, polypropylene (PP)/polystyrene (PS) [17], polyethylene (PE)/PS [14] and ethylene-α-octene copolymer (POE)/PS [13] blend pellets have been prepared with this method and processed into dumbbell-shaped bars or rods by melt processing. Although PS particles are rigid and are generally considered to be non–compatible with PP, PE, and POE, all the blends have a good overall performance in regard to stiffness, strength, ductility, and toughness because of their small size and the existence of grafting which results in good interfacial adhesion between the dispersed phase and the matrix phase. For example, the yield strength of a PP/PS blend containing 18% PS is 27% higher than that of neat PP while the elongation at break is still over 300% [17]. 

Polyamide 6 (PA6) is an important engineering plastic [20,21,22,23], and PS is a widely used commodity plastic. To take the advantages of both PA6 and PS and overcome the shortages of single polymers, PA6/PS blends have been extensively investigated [24,25,26,27,28,29,30,31,32,33,34,35,36,37]. PA6 and PS are non-compatible due to the large difference in polarity, and PA6/PS blends obtained by traditional melt mixing have very poor mechanical properties [30,31]. Therefore, PA6/PS blends are often prepared by reactive compatibilization in the presence of a reactive compatibilizer, whereby a block or graft copolymer is formed at the interface of PA6/PS during melt compounding [25,31,32,33,34,35]. The improvement in toughness of PA6/PS blends is usually achieved by the addition of an elastomer or suitable compatibilizer, although this often leads to a reduction in stiffness or strength [34,36,37].

Recently, we reported the preparation of PA6/PS quasi-nanoblend pellets with an average PS particle size of about 100 nm using diffusion and subsequent polymerization of styrene in commercial PA6 pellets [26]. Although styrene can barely diffuse into dry PA6 due to the small free volume of PA6, the diffusion paths can be opened up by presorbing water, because the sorbed water drastically decreases the glass transition temperature (Tg) and increases the free volume of PA6. Consequently, styrene can diffuse into wet PA6 pellets by replacing free water in the neat styrene medium. The goal of this work is to investigate the morphology and mechanical properties of the PA6/PS quasi-nanoblend pellets prepared by this unique method. Thus, the pellets are prepared on a much bigger scale and processed into cuboid-shaped bars by injection molding. Compared to neat PA6, the toughness of the blend is significantly improved without any loss of stiffness, strength, or ductility, as shown by impact, tensile, and flexural tests. The good mechanical properties of such PA6/PS blends are correlated with their fine particle sizes and the occurrence of PS grafting onto PA6 during the preparation of the blend pellets and injection molding. PS distribution in bars, water-sorption of bars, dimensional stability of bars against water, and the degree of crystallinity of PA6 are also investigated in the current study.

## 2. Materials and Methods

**Materials.** Commercial grade PA6 pellets (1013B) were sourced from Ube Industries, Ltd., Tokyo, Japan, and dried at 110 °C for 24 h before use. Styrene was purified by vacuum distillation. Benzoyl peroxide (BPO) was purified twice by recrystallization from chloroform/methanol. Other chemicals were of analytical grade and were used as received.

**Preparation of PA6/PS blend pellets.** Eight hundred grams of PA6 pellets and 600 mL of deionized water were added to a 2000 mL flask equipped with a condenser and a mechanical stirrer. The mixture was heated with a water bath at 90 °C for 3 h under stirring. The pellets were separated by vacuum filtration and wiped with filter papers, then transferred into a new flask containing 600 mL of neat styrene, and the diffusion of styrene proceeded at 90 °C for 1.5 h. The pellets were again separated by vacuum filtration and transferred into a new flask which contained 600 mL of deionized water. With the aid of chloroform to initiate polymerization, 2.4 g of BPO was added, and the mixture was stirred at 90 °C for 3 h. The blend pellets were dried in a vacuum oven at 110 °C to a constant weight.

**Injection–molding of PA6/PS blend pellets.** After drying, the PA6/PS blend pellets were injection-molded into cuboid-shaped bars of 80 × 10 × 4 mm^3^ (length × width × thickness) in an injection molding machine (HT-125, Haitian Plastics Machinery, Ningbo, China), with a temperature profile of 230, 230, 220, 220, and 210 °C from the feeding zone to the nozzle. The injection pressure was 50 bar, and the injection speed was 40 mm/s. For comparison, neat PA6 pellets were injection-molded similarly.

**Characterization.** The PS content in the PA6/PS blend pellets was defined as the weight ratio of PS to PA6 and determined by the ATR-FTIR method. PA6/PS pellets were hot-pressed into a thin film of 100 μm thickness. The ratio of the peak areas of the absorptions at 758 cm^−1^ (PS) and 1645 cm^−1^ (PA6), A_1_/A_2_, was determined and the PS content was obtained (7.3%) using a previously-reported calibration curve [27].

The percentage of PS grafting is defined as the weight ratio of grafted PS to PA6 and was measured as follows: PA6/PS pellets were hot-pressed into thin slices and thoroughly extracted with chloroform to remove the ungrafted PS and then analyzed by ATR-FTIR. The ratio of A_1_/A_2_ was determined, and the percentage of PS grafting was obtained (2.0%) using the calibration curve.

The diametrical distribution of PS in the blend pellets and the PS distribution along the width direction of the blend bars were investigated using Microscopic Fourier Transform Infrared Spectroscopy (Micro-FTIR) (model Nicolet 6700, Thermo Electron Corporation, Madison, WI, USA), with a microsampling area of 100 × 100 μm^2^ and a step length of 100 μm. For sample preparation, thin slices were cut through the center of a blend pellet and the cross section of a bar. A_1_/A_3_, defined as the ratio of the peak areas of absorption at 758 cm^−1^ and 1122 cm^−1^, which are the characteristic peaks of PS and PA6, respectively, was taken as a measure for the PS/PA6 ratio at the measured position.

A JSM-7401 field emission scanning electron microscope (FESEM) (JEOL, Tokyo, Japan) at an accelerating voltage of 3 kV was used to investigate the morphology of the PS/PA6 blend pellets and bars. The pellet was cut into two symmetrical parts and stirred in aqueous trichloroacetic acid (15%) for 30 min to remove the amorphous phase of PA6 of the cross section, washed ultrasonically in deionized water for 5 min, and dried in an oven. The PA6/PS bars were cryofractured in liquid nitrogen. All surfaces were gold sputtered before FESEM observation.

Differential scanning calorimetry (DSC) experiments were conducted with a TA Instrument (model Q100, TA Instrument, Newcastle, DE, USA). Samples of about 6 mg were placed in a standard sealed aluminum pan and then heated from 40 to 240 °C at a constant rate of 10 °C/min under a constant flow of nitrogen (50 mL/min). The melting temperature ($T_{m}$) was defined as the maximum of the melting peak, and the enthalpy of melting ($\Delta H_{m}$) was calculated as the integral of the melting peak, corrected by the weight percent of PA6 in the blend.

Water sorption and dimensional changes of the bars were carried out by comparing wet and dry samples. The wet and dry samples refer to those stored in a container conditioned at 70% relative humidity (RH) for 5 days and those dried in a vacuum oven at 110 °C for 24 h, respectively. The weights of the bars before and after sorption of water were recorded. Water sorption was defined as the weight ratio of absorbed water/dry sample. The lengths, widths and thicknesses of the bars were measured by Vernier calipers.

Tensile property testing was performed on a GT-TCS 2000 tensile tester (Gotech, Taichung, Taiwan) with a crosshead speed of 10 mm/min, with a distance of 3 cm between fixtures. Both dry and wet samples were tested. Data from at least three parallel measurements were averaged. The values of the Young’s modulus were used for the slopes of the stress–strain curves at an elongation range of 3–7%.

Flexural tests were conducted on a MTS-CMT 6104 universal mechanical tester (MTS, Eden Prairie, MN, USA), in accordance with ISO 178: 2001. Notched Izod impact tests were carried out with the MTS-ZBC 7151-B Impact Tester (MTS, Eden Prairie, MN, USA), in accordance with ISO179-2: 2000. 

## 3. Results and Discussion

### 3.1. Distribution of PS and Morphology of PA6/PS Pellets and Molded Bars

PA6 and PS are non-compatible. Further, the maximum PS content in PA6/PS blend pellets prepared by the method of diffusion and subsequent polymerization of styrene in commercial PA6 pellets in the previously reported small-scale procedure was only 6.7% [26]. Thus, in this work, the conditions for obtaining the maximum PS content were used to carry out large-scale preparation (40 times) in order to obtain sufficient PA6/PS blend pellets for injection molding and evaluating the mechanical properties of this unique blend. However, the apparent concentrations of PA6 pellets in each step, i.e., the ratios of pellet/medium, were 2.7 times those used in the previously reported small-scale procedure. Although the medium water in the water-sorption step and the medium styrene in the diffusion step were also the diffusants, the variation in the pellet/medium ratio should not have any effect on the PS content, PS distribution, or blend morphology because only a tiny amount of the medium was able to diffuse into the pellets in each step.

**Figure 1:**
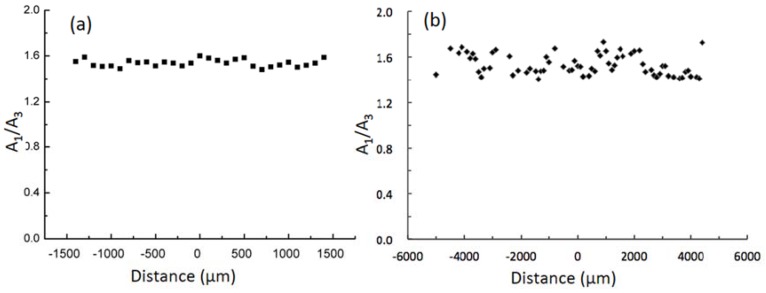
Micro-FTIR profiles of polyamide 6/polystyrene (PA6/PS): (**a**) pellet along the diameter direction and (**b**) bar along the width direction (distance 0 is the center of the pellet or bar and A_1_/A_3_ is the ratio of the peak areas of the absorptions at 758 cm^−1^ and 1122 cm^−1^, representing PS/PA6 at the measured position).

The distribution of PS in both blend pellets and bars was investigated by Micro-FTIR for A_1_/A_3_ (i.e., the ratio of the peak areas of absorption at 758 cm^−1^, a characteristic peak of PS, and 1122 cm^−1^, a characteristic peak of PA6), as a measure of the PS/PA6 ratio at each measured position. As shown in Figure 1a, the PS distribution along the diameter direction of a pellet was almost homogeneous and the average value of A_1_/A_3_ was slightly higher than that in the small-scale preparation [26], indicating a slightly higher PS content. ATR-FTIR was used to measure the PS content using a previously-reported calibration curve [27] and the value was 7.3%, slightly higher than that of the small-scale procedure, and basically in agreement with the Micro-FTIR results. As shown in Figure 1b, the distribution of PS along the width direction of a molded bar was basically homogeneous with the A_1_/A_3_ values of around 1.5, which is practically the same as that of the pellets.

**Figure 2:**
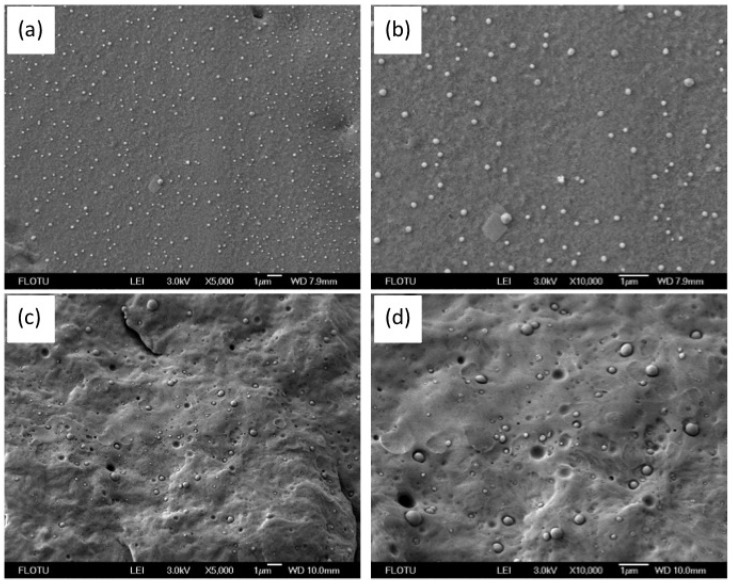
Field emission scanning electron microscope (FESEM) images of (**a**,**b**) a cross section of the PA6/PS blend pellet after etching with 15% aqueous trichloroacetic acid and (**c**,**d**) the cryofractured surface of the PA6/PS bar at two different magnifications.

FESEM was used to investigate the phase morphology of both the blend pellets and the bars and the resulting micrographs are shown in Figure 2. The cross section of a pellet was observed after etching the amorphous PA6 portions with aqueous trichloracetic acid for better observation, since PS particles could not be observed clearly without removing the amorphous PA6. As shown in Figure 2a,b, a typical sea-island structure was observed and spherical PS particles were evenly distributed in the blend pellet. The average particle size was 130 nm, slightly higher than that in the small-scale procedure (103 nm). As shown in Figure 2c,d, the sizes of the PS particles were still small in the molded bar, but the particle size distribution was wider than that in the blend pellet. Almost all the PS particles were of nano or submicron sizes. The biggest particle size was around 700 nm and the average particle size was 240 nm, indicating that only slight coalescence of PS particles takes place during injection molding. The small PS particle size should be beneficial for the improvement of mechanical properties.

### 3.2. Crystallization Behavior of PA6 in PA6/PS Pellets and Molded Bars

**Figure 3:**
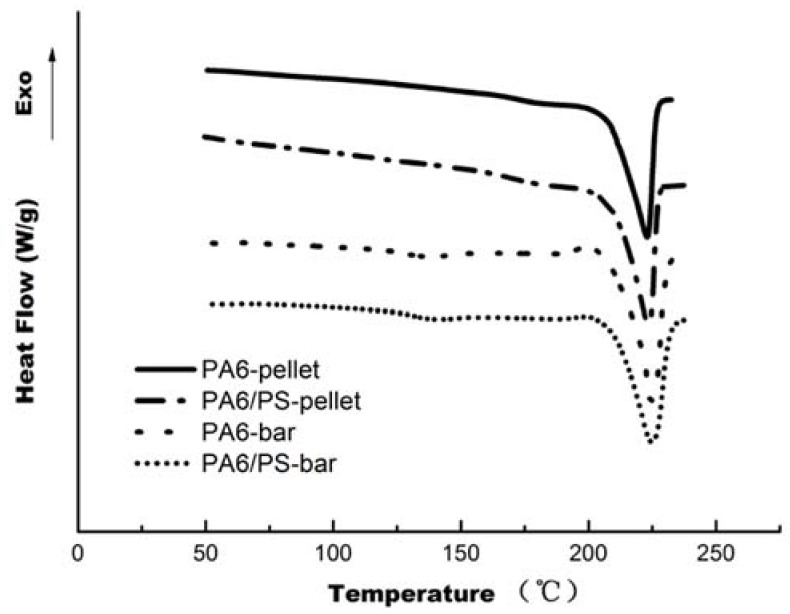
DSC first heating curves of PA6 and PA6/PS pellets and bars at a heating rate of 10 °C/min.

DSC was used to investigate the crystallization behavior of PA6 in PA6/PS pellets and molded bars. Typical DSC heating curves are shown in Figure 3, and the melting temperature ($T_{m}$) and enthalpy of melting ($\Delta H_{m}$) data are listed in Table 1. The $T_{m}$ and $\Delta H_{m}$ of PA6 in the blend pellets were almost unchanged compared to those of neat PA6 pellets, confirming that the diffusion and subsequent polymerization of styrene only occurs in the amorphous regions of PA6 pellets, which is the basic principle of the method of diffusion and subsequent polymerization of a monomer (or monomer mixture) in polymer pellets.

The $T_{m}$ and $\Delta H_{m}$ of PA6 in the injection-molded PA6/PS bars were also similar to those of neat PA6 bars, indicating that the presence of PS in the blend has almost no effect on the crystallization behavior of PA6, and the degree of crystallinity is not the reason for the good overall mechanical properties of PA6/PS blend bars.

### 3.3. Water Sorption and Dimensional Changes of Injection Molded PA6/PS Blend Bars

The resistance of injection-molded PA6/PS bars to water was assessed and compared with that of neat PA6 bars by placing the bars in 70% RH for 5 days. The water sorption of PA6/PS bars decreased to 2.23%, equivalent to 2.41% when referred to the PA6 fraction. Considering possible experimental error, the latter value is closed to water sorption of neat PA6 bars (2.54%), indicating that the decrease in water sorption of PA6/PS bars is solely due to incorporation of the hydrophobic PS fraction. This reveals that the homogeneous incorporation of PS is less efficient in preventing the sorption of water than the PS-rich shell structure reported earlier, in which a PS content of 1.2% can significantly lower water sorption [27].

**Figure 4:**
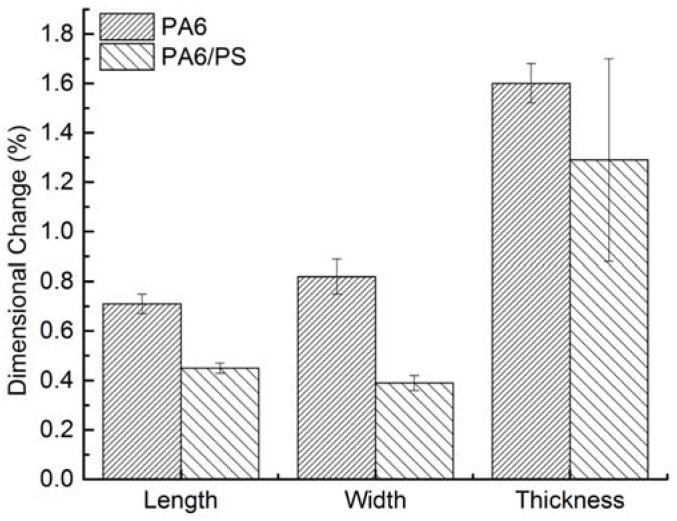
Dimensional changes of PA6 and PA6/PS bars after storage in 70% RH for 5 days.

The dimensional expansion in the three directions of the bars upon sorption of water was measured, and the results are shown in Figure 4. Clearly, PA6/PS bars show better dimensional stability than neat PA6 bars in all three directions because the decreased water sorption resulted from the presence of hydrophobic PS. The volume expansion of PA6/PS bars caused by water sorption was 2.41%, significantly lower than that of neat PA6 bars (3.16%).

### 3.4. Mechanical Properties of Injection Molded PA6/PS Blend Bars

Tensile, flexural, and notched impact tests were used to evaluate the mechanical properties of PA6/PS bars injection-molded from PA6/PS blend pellets, and the results are summarized in Table 2. The tensile tests were carried out on both dry and wet samples, while the flexural and notch impact tests were only carried out on dry samples.

**Figure 5:**
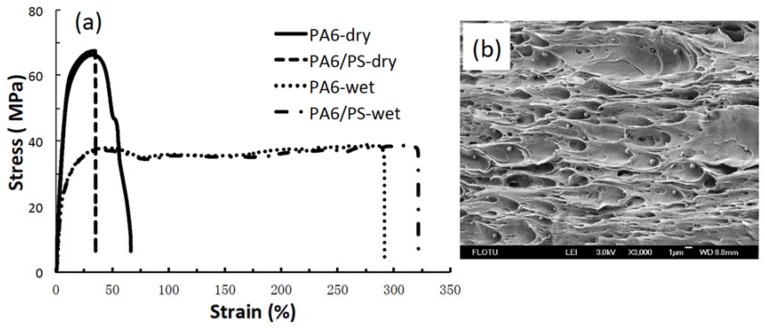
(**a**) Typical stress–strain curves of PA6 and PA6/PS bars in both dry and wet states and (**b**) FESEM micrograph of the necked part of PA6/PS wet bars after the tensile test, showing unstretched PS particles.

The typical stress–strain curves of PA6/PS bars along with those of neat PA6 bars are shown in Figure 5a. The tensile behaviors of PA6/PS bars were very similar to those of neat PA6 bars in both dry and wet states, indicating that the presence of immiscible PS particles does not cause any deterioration of the tensile properties of PA6 even in the wet state, although no compatibilizer was used. The good ductility of wet PA6/PS bars suggests that the PA6/PS interface is not damaged upon sorption of water because good interfacial adhesion resulted from the small particle size and the occurrence of PS grafting onto PA6 during the preparation of the blend pellets and injection molding. The necking region of the elongated PA6/PS wet bars was observed by FESEM along the stretching direction, and the micrograph is shown in Figure 5b. PS particles were not stretched and did not act as stress concentrators because of their small size and good interfacial adhesion [17]. For the same reason, the flexural strength was also retained (Table 2).

Also, as seen in Table 2, the notched impact strength of PA6/PS bars had a remarkable increase of 41.8% compared to that of neat PA6, achieving efficient toughening, although PS is brittle. This occurs because the interfacial adhesion is good enough to ensure stress transfer from the matrix to the particles, and more importantly, the large number of small PS particles generates a huge interfacial area that can effectively dissipate impact energy.

It has been reported that a PA6/PS (9/1, *v*/*v*) blend prepared in a twin-screw extruder at a screw speed of 100 rpm with a draw-down ratio of 1 can significantly reduce the tensile modulus and strength compared to neat PA6 [31]. The PA6/PS (9/1, *w*/*w*) bars injection molded from a compound prepared in a twin-screw extruder at 500 rpm have a slightly decreased tensile strength and almost unchanged toughness probably because of the much smaller PS particle size (1.5 μm) [38]. Even in the presence of 5 wt % of ethene–propene elastomer, the toughness of PA6/PS/EPR (9/5/5, *w*/*w*/*w*) only increases by 18% compared to neat PA6 [34]. The remarkable 41.8% increase in toughness with a maintained stiffness, strength, and ductility achieved in the PA6/PS (100/7.3, *w*/*w*) blend prepared in this work clearly demonstrates the advantage of fine PS particle size and good interfacial adhesion.

## 4. Conclusions

PA6/PS bars, injection molded from PA6/PS blend pellets, prepared by diffusion and subsequent polymerization of styrene in commercial PA6 pellets were shown to have significantly increased toughness with maintained stiffness, strength, and ductility compared to neat PA6 bars, because only limited coalescence occurs during injection molding. The small particle sizes and good interfacial adhesion between PS particles and the PA6 matrix resulted from the occurrence of PS grafting onto PA6 during the preparation of the blend pellets, and injection molding was able to ensure effective stress transfer from the matrix to particles and dissipate impact energy. PA6/PS bars were also shown to have an improved antiwater ability and dimensional stability due to the presence of hydrophobic PS.

This work proves that the PA6/PS quasi-nanoblend pellets prepared by diffusion and subsequent polymerization of styrene in polyamide 6 pellets are useful stock for plastic processing, and further confirms that diffusion and subsequent polymerization of a monomer (or monomer mixture) in polymer pellets is a good method for preparing polymer blend pellets in preparation for low-shear manufacturing processes such as injection molding.

## Figures and Tables

**Table 1:** Differential scanning calorimetry (DSC) results of PA6 and PA6/PS pellets and bars.

	$T_{m}/{}^{\circ}C$	$\Delta H_{m}/\left(J/g\right)$
PA6 Pellet	223.0	66.1
PA6/PS Pellet	223.5	69.1
PA6 Bar	224.7	77.1
PA6/PS Bar	225.0	73.3

**Table 2:** Mechanical properties of PA6 and PA6/PS bars.

	Young’s Modulus/MPa	Tensile Strength/MPa	Elongation at Break/%	Flexural Strength/MPa	Notched Impact Strength/(KJ/m^2^)
PA6-dry	281.0 ± 15.2	66.4 ± 0.0	42.9 ± 29.2	84.3 ± 0.5	5.5 ± 0.3
PA6/PS-dry	278.4 ± 4.7	67.7 ± 0.3	34.8 ± 0.5	88.3 ± 0.3	7.8 ± 0.7
PA6-wet	186.6 ± 15.9	38.0 ± 0.7	295.4 ± 21.9	-	-
PA6/PS-wet	192.9 ± 18.8	37.1 ± 0.9	293.9 ± 39.4	-	-
